# Supplementary material for: Influence of the Ethanol Content of Adduct on the Comonomer Incorporation of Related Ziegler–Natta Catalysts in Propylene (Co)polymerizations
Source: Polymers (Basel). 2023 Nov 21;15(23):4476. doi: 10.3390/polym15234476 (PMC10708330; doi:10.3390/polym15234476)
Supplement: Supplementary file 1 [file polymers-15-04476-s001.zip › polymers-2673478-supplementary.pdf]

Influence of the Ethanol Content of Adduct on the Comonomer Incorporation of Related Ziegler–Natta Catalysts in Propylene (Co)polymerizations

Mohammadreza Mehdizadeh [1], Fereshteh Karkhaneh [1,*], Mehdi Nekoomanesh [1], Samahe Sadjadi [1], Mehrsa Emami [1], HamidReza Teimoury [2], Mehrdad Salimi [2], Miquel Solà [3], Albert Poater [3,*], Naeimeh Bahri-Laleh [1,*] and Sergio Posada-Pérez [3,*]

[1] Iran Polymer and Petrochemical Institute (IPPI), Tehran 14965/115, Iran
[2] Research & Development Center, Kermanshah Polymer Company, Kermanshah 14965/115, Iran
[3] Institut de Química Computacional i Catàlisi and Departament de Química, Universitat de Girona, c/ Maria Aurèlia Capmany 69, 17003 Girona, Spain
* Correspondence: f.karkhaneh@ippi.ac.ir (F.K.); albert.poater@udg.edu (A.P.); n.bahri@ippi.ac.ir (N.B.-L.); sergio.posada@udg.edu (S.P.-P.)

Characterization

X-ray diffraction (XRD) analysis of the catalysts was carried out by using a Siemens D-5000 X-ray diffractometer (USA) at 40 kV and 25 mA with a copper target (λ = 1.54 A) and a scanning rate of 3 °/min. The results were analyzed by X'pert Highscore Plus software. SEM images of the synthesized catalysts and also EDX analysis and elemental maps were recorded employing an SEM instrument (SEM model S-3000 N, Hitachi, Japan). Thermogravimetric analysis (TGA) was employed to explore the thermal stability of the adduct samples in the range of 25–400 °C by utilizing TGA instrument (Mettler Toledo Inc., Switzerland) under N_2 atmosphere and with the heating rate of 10°C/min. The adduct samples showed two step weight loss in the range of 150-250 °C, which is attributed to the EtOH desorbtion from the adduct samples.[i] By calculating the weight loss between the aforementioned temperatures, the ethanol content was determined. The specific surface area of the catalysts was determined through Brunauer–Emmett–Teller (BET) examinations using a BELSORP Mini II instrument. Particle sizes of the synthesized catalyst and their distributions were measured utilizing a Malvern Zetasizer Nano ZS instrument (United Kingdom) according to ISO 13320-2 standard.

The DSC thermograms of the synthesized polymer samples were recorded employing DSC Mettler Toledo (Switzerland), under N_2 atmosphere at a heating rate of 10°C/min. the degree of crystallinity (X_c) and melting temperature (T_m) were achieved by using a second heating scan, and X_c values were obtained using the following formula:

$$X_C = \frac{\Delta H_m}{\Delta H_m^{+}} \quad \text{(S1)}$$

Where, ΔH^{+}_{m} is the specific melting enthalpy of 100% crystalline polypropylene (2090 J/g),[ii] and ΔH_m is the specific melting enthalpy of the prepared polymers. The thickness of lamellae and their distribution in the synthesized polymers were analyzed via successive self-nucleation and annealing (SSA) technology. The SSA analysis was carried out by a Mettler Toledo instrument (Switzerland) according to the Muller method[iii,iv] as the following steps: (I) heating the sample from 50 to 200°C and holding it at this temperature for 5 min to delete thermal history, (II) cooling from 200 to 50°C and holding at this temperature for 5 min (III) heating from 50 °C to T_s=164 °C and holding for 5 min, (IV) cooling from T_s=164 °C to 50°C, (V) repeating steps 3 and 4 with new lower Ts, ranging from 164 to 144 °C, with ΔT_s = 5 °C intervals, (VI) finally heating the PP sample from 50 to 200°C. The heating and cooling rate in all runs was 10°C/min. The lamellar thickness was obtained from SSA curves employing the Thomson-Gibbs equation.[v]

$$T_m = T_m^{\circ}\left(1 - \frac{2\sigma}{\Delta \mathrm{H}\, \mathrm{l}_c}\right) \quad \text{(S2)}$$

Where l_c is the lamellar thickness, T_m is the middle temperature of each melt peak at the SSA curve, T°_{m} is the melting point of an infinite PP crystal (460 K),[vi,vii] ΔH is the heat of fusion of the crystal with an infinite l_c (184 × 106 J/m^3), and σ is the free energy of the lamellar surface (0.0496 J/m^2).

To elucidate the chemical composition distribution of polymer samples, a homemade P-TREF (Temperature Rising Elution Fractionation) consisting of a cylindrical steel column with a 7 cm diameter and 40 cm height has been employed, packed with sands with the average diameter of 700 microns. A procedure described by Kebritchi *et al.*[viii] was used to perform the TREF analyses,[ix] packed with sands with the average diameter of 700 microns. This column was submerged in an oil bath equipped with a programmable thermostat to set the temperature at crystallization and elution cycles. In each fractionation run, 600 cc of polymer solution (1 wt. % in xylene containing 0.05 wt. % of irganox 1010 as antioxidant) at 140 °C was injected to the column. It was kept at this temperature for 2 hours and then cooled to the room temperature with the rate of 2 °C/hr to crystallize. Then the first fraction (xylene soluble) was washed through the column with fresh xylene at room temperature. For the next fraction, column temperature was raised to the desired temperature (here 40°C) at the rate of 0.5 °C/min. kept at that temperature for 2 hours and washed with fresh xylene at 40 °C. This procedure was repeated for all fractions.

HNMR analysis was conducted to shed light on the donor type utilizing a Bruker DRX400MHz NMR spectrometer in deuterated DMSO as solvent.

Steric maps

Without taking into account the hydrogen atoms in the steric maps:

TS-DEP-re

%V Free	%V Buried	% V Tot/V Ex
57.3	42.7	99.9

Quadrant	V f	V b	V t	%V f	%V b
SW	70.4	60.4	130.8	53.8	46.2
NW	73.3	57.5	130.8	56.0	44.0
NE	82.5	48.3	130.8	63.1	36.9
SE	73.8	57.0	130.8	56.4	43.6

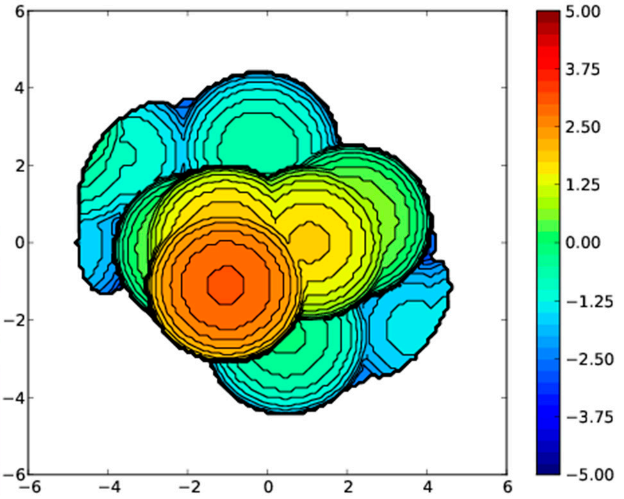

TS-DEP-si

%V Free	%V Buried	% V Tot/V Ex
51.7	48.3	99.9

Quadrant	V f	V b	V t	%V f	%V b
SW	60.7	70.1	130.8	46.4	53.6
NW	69.2	61.6	130.8	52.9	47.1
NE	83.8	47.0	130.8	64.0	36.0
SE	57.1	73.7	130.8	43.7	56.3

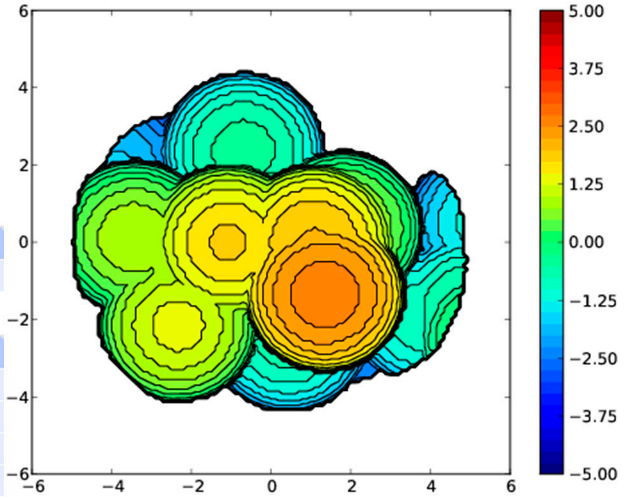

TS-DIBP-re

%V Free	%V Buried	% V Tot/V Ex
57.4	42.6	99.9

Quadrant	V f	V b	V t	%V f	%V b
SW	82.6	48.2	130.8	63.1	36.9
NW	73.8	57.0	130.8	56.4	43.6
NE	70.5	60.4	130.8	53.9	46.1
SE	73.4	57.4	130.8	56.1	43.9

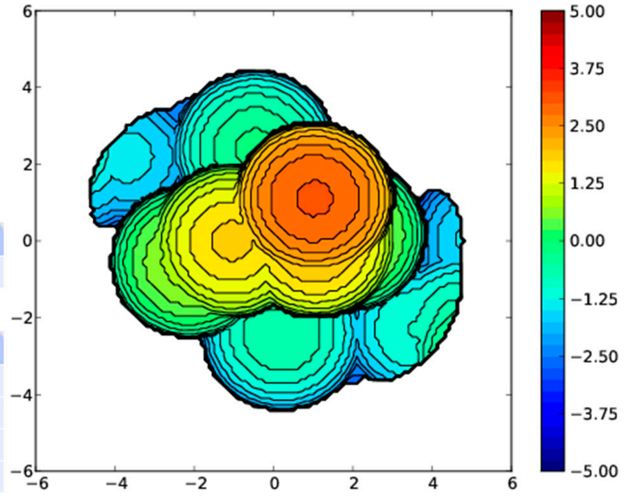

TS-DIBP-si

%V Free	%V Buried	% V Tot/V Ex
51.8	48.2	99.9

Quadrant	V f	V b	V t	%V f	%V b
SW	60.7	70.1	130.8	46.4	53.6
NW	69.2	61.6	130.8	52.9	47.1
NE	84.2	46.6	130.8	64.3	35.7
SE	56.8	74.0	130.8	43.4	56.6

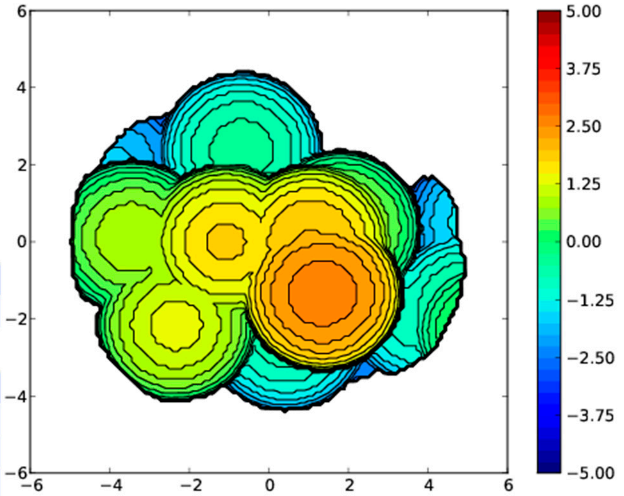

With taking into account the hydrogen atoms in the steric maps:

TS-DEP-re

%V Free	%V Buried	% V Tot/V Ex
55.3	44.7	99.9

Quadrant	V f	V b	V t	%V f	%V b
SW	79.7	51.1	130.8	61.0	39.0
NW	71.1	59.7	130.8	54.4	45.6
NE	66.4	64.4	130.8	50.8	49.2
SE	71.9	58.9	130.8	55.0	45.0

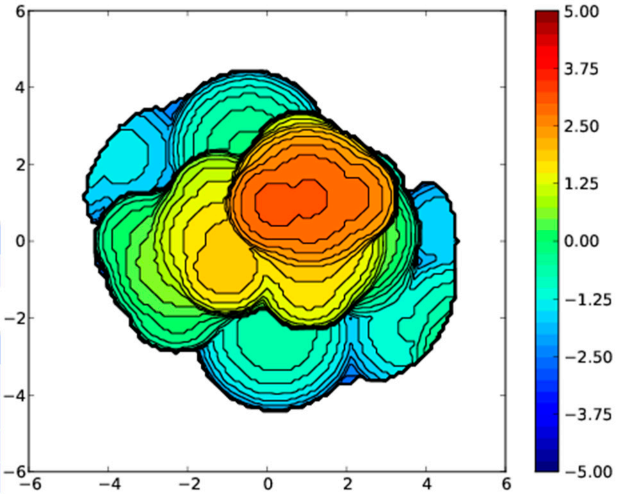

TS-DEP-si

%V Free	%V Buried	% V Tot/V Ex
49.5	50.5	99.9

Quadrant	V f	V b	V t	%V f	%V b
SW	56.2	74.6	130.8	43.0	57.0
NW	66.9	63.9	130.8	51.2	48.8
NE	81.1	49.7	130.8	62.0	38.0
SE	54.7	76.1	130.8	41.8	58.2

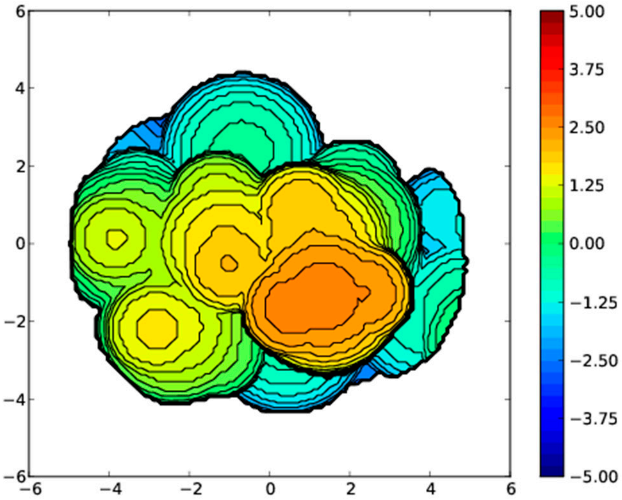

TS-DIBP-re

%V Free	%V Buried	% V Tot/V Ex
55.4	44.6	99.9

Quadrant	V f	V b	V t	%V f	%V b
SW	80.1	50.7	130.8	61.2	38.8
NW	71.2	59.7	130.8	54.4	45.6
NE	66.5	64.3	130.8	50.8	49.2
SE	72.0	58.8	130.8	55.1	44.9

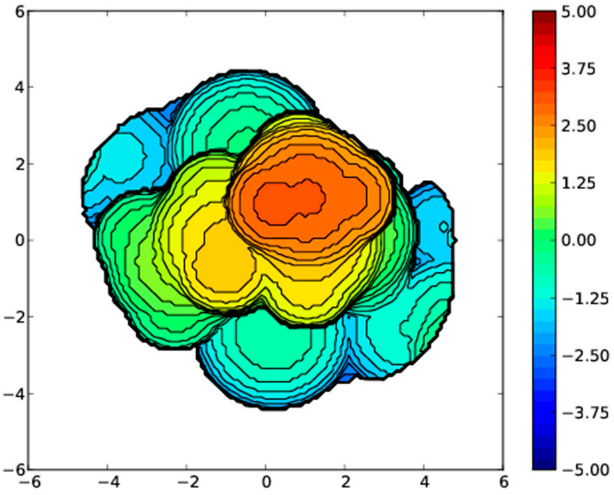

TS-DIBP-si

%V Free	%V Buried	% V Tot/V Ex
49.5	50.5	99.9

Quadrant	V f	V b	V t	%V f	%V b
SW	56.2	74.6	130.8	43.0	57.0
NW	66.9	63.9	130.8	51.2	48.8
NE	81.5	49.3	130.8	62.3	37.7
SE	54.4	76.4	130.8	41.6	58.4

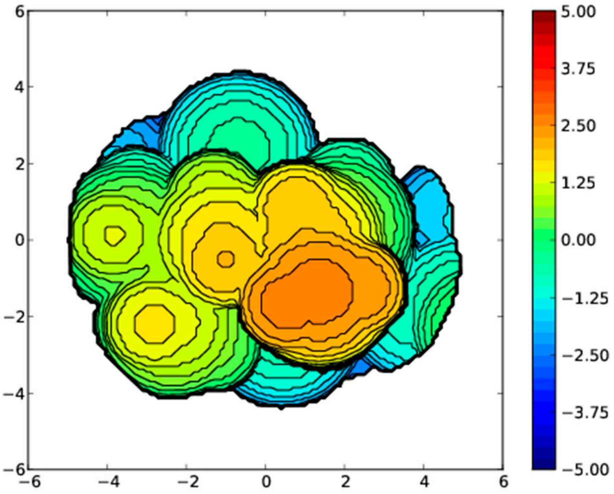

Figure S1. $\%V_{Bur}$ and topographic steric maps in Å (XY plane) of the transition states (re and si) for DEP and DIBP using the central point between the two carbon atoms that form the C-C bond, the titanium the z axis and the linking C atom of the entering olefin to define the xz plane (in Å).

Table S1. Xyz coordinates and energies (in atomic units) of all the computed species.

121

I_DEP SCF Done: -20274.2390693 [illegible].U.

Mg	3.264258	-2.635110	-0.623863
Cl	5.103161	-2.452636	-2.194599
Mg	6.433134	-0.850260	-0.964275
Cl	8.447801	-0.615044	-2.315357
Mg	9.422588	1.319779	-1.250624
Cl	11.467628	1.027553	-0.324706
Ti	-0.040744	-4.288082	0.099708
Cl	-1.674393	-4.144598	1.935442
C	-0.180118	-6.373100	0.076788
H	-0.531701	-6.788374	1.030390
C	-1.259459	-6.179711	-0.967857
H	0.732723	-6.863400	-0.285719
H	-1.418987	-5.046755	-1.131727
Cl	1.703079	-4.277021	-1.613026
Mg	-3.206785	-2.512928	0.935134
Cl	-5.078057	-2.213537	2.450548
Mg	-6.353080	-0.691269	1.069641
Cl	-8.427355	-0.389610	2.315096
Mg	-9.319419	1.483180	1.089062
Cl	-8.387827	3.540378	1.956208
Mg	-6.215782	3.225009	0.961585
Cl	-5.164483	5.354586	1.618917
Mg	-3.067190	5.025441	0.524235
Cl	-1.875664	7.155547	1.075927
Mg	0.099012	6.558525	-0.080762
Cl	-1.168329	4.853200	-1.319237
Cl	-1.328464	-2.460271	-1.058959
Cl	1.351356	-2.542573	1.275197

Cl	-11.349999	1.269254	0.108670
Cl	1.344134	4.887737	1.227161
Cl	2.077275	7.070099	-1.270820
Mg	3.232853	4.940398	-0.651303
Cl	5.305899	5.182370	-1.809292
Mg	6.340775	3.070599	-1.075965
Cl	8.476105	3.335493	-2.171655
Cl	1.935169	3.095909	-1.729123
Cl	-1.775938	-0.505027	1.832889
Mg	0.050140	-0.662357	0.097268
Cl	1.860307	-0.639428	-1.649151
Cl	-1.809661	3.219106	1.711852
Mg	0.072347	3.002434	0.004234
Cl	-1.217879	1.212175	-1.131229
Cl	1.343893	1.240301	1.223099
Mg	-3.121882	1.267126	0.598472
Cl	-4.276279	-0.578194	-0.530497
Cl	-5.044422	1.443707	2.207752
Cl	-4.255813	3.161695	-0.766732
Mg	3.234531	1.178746	-0.539888
Cl	4.414334	3.129379	0.706831
Cl	5.118924	1.239230	-2.193707
Cl	4.383099	-0.606316	0.681720
Cl	7.455534	1.284438	0.418082
Cl	-7.300701	1.351697	-0.474549
C	-6.048898	-1.547454	-3.771791
C	-6.175889	-2.275225	-2.580269
C	-4.919624	-1.708624	-4.577989
H	-4.815568	-1.115679	-5.487921
C	-5.167350	-3.192336	-2.212288
C	-3.912743	-2.602377	-4.202585

H	-3.016178	-2.712676	-4.814518
C	-4.037977	-3.343926	-3.024627
H	-3.249425	-4.032287	-2.717227
H	-6.818531	-0.819660	-4.029818
C	-7.270882	-1.941974	-1.634924
C	-5.179011	-3.997639	-0.950730
O	-7.097409	-1.951817	-0.400156
O	-4.250197	-3.889501	-0.141309
O	-8.411382	-1.646684	-2.224508
C	-9.549925	-1.139585	-1.406316
H	-9.336529	-1.329567	-0.345508
H	-9.565721	-0.057261	-1.583034
C	-10.817086	-1.812491	-1.877611
H	-11.654794	-1.358464	-1.328685
H	-10.977905	-1.652584	-2.952872
H	-10.799686	-2.892253	-1.671859
O	-6.097444	-4.918228	-0.696195
C	-7.217390	-5.233685	-1.597027
H	-7.165852	-4.609794	-2.499943
H	-7.057505	-6.282296	-1.885491
C	-8.513982	-5.049440	-0.830976
H	-9.361588	-5.302779	-1.484880
H	-8.542447	-5.704698	0.049901
H	-8.627976	-4.012288	-0.489697
C	6.340978	-1.280487	3.878786
C	6.382713	-2.138462	2.771581
C	5.228830	-1.280522	4.724450
H	5.191303	-0.590440	5.568773
C	5.306087	-3.018988	2.526525
C	4.154619	-2.137296	4.469186
H	3.272541	-2.121342	5.111289

C	4.193357	-3.006025	3.374549
H	3.349893	-3.663640	3.161777
H	7.161451	-0.579763	4.035683
C	7.449590	-1.966087	1.757136
C	5.255937	-3.960409	1.364468
O	7.216633	-2.057681	0.535506
O	4.315374	-3.929964	0.562074
O	8.639390	-1.697360	2.259073
C	9.728027	-1.384620	1.291354
H	9.282047	-0.829373	0.462142
H	10.394951	-0.721353	1.849968
O	6.162872	-4.916981	1.204503
C	7.247204	-5.142404	2.174891
H	8.071383	-4.462316	1.912994
H	6.888490	-4.898137	3.185748
C	7.662828	-6.592765	2.043478
H	8.497846	-6.795277	2.730420
H	6.831111	-7.264125	2.297857
H	7.993095	-6.811884	1.019032
C	10.400636	-2.645653	0.797019
H	10.809536	-3.238705	1.627776
H	9.703635	-3.257167	0.206457
H	11.229385	-2.344656	0.139537
C	-2.648663	-6.666931	-0.527548
H	-3.414447	-6.409730	-1.276487
H	-2.631322	-7.761669	-0.417599
H	-2.943230	-6.215343	0.428518
C	-0.875113	-6.695595	-2.363608
H	0.104339	-6.308989	-2.675927
H	-0.826382	-7.794875	-2.344073
H	-1.621776	-6.398983	-3.117295

145

I_DIBP SCF Done: -20588.8764755 □.U.

Mg	3.267063	-2.319843	-0.672709
Cl	5.041612	-2.139874	-2.320551
Mg	6.400299	-0.527816	-1.141234
Cl	8.422932	-0.271890	-2.503861
Mg	9.398527	1.622884	-1.353049
Cl	11.461041	1.447188	-0.445873
Ti	-0.026643	-3.982821	0.110094
Cl	-1.643926	-3.844072	1.960492
C	-0.153377	-6.068696	0.099769
H	-0.497184	-6.480646	1.057586
C	-1.239412	-5.887940	-0.940060
H	0.760478	-6.555610	-0.264908
H	-1.403723	-4.757114	-1.114427
Cl	1.692445	-3.970966	-1.626760
Mg	-3.194774	-2.201649	1.002926
Cl	-5.000300	-1.921760	2.595376
Mg	-6.330974	-0.438411	1.229296
Cl	-8.360070	-0.054799	2.546356
Mg	-9.291475	1.793838	1.282764
Cl	-8.332251	3.853208	2.098862
Mg	-6.183699	3.496253	1.079389
Cl	-5.131221	5.640270	1.711897
Mg	-3.061587	5.321732	0.572961
Cl	-1.863292	7.457181	1.093639
Mg	0.089454	6.859983	-0.097574
Cl	-1.199152	5.147666	-1.306349
Cl	-1.341938	-2.161212	-1.030897
Cl	1.378424	-2.232576	1.259273
Cl	-11.329651	1.622075	0.318838

Cl	1.361723	5.194897	1.194662
Cl	2.045912	7.369887	-1.322984
Mg	3.217037	5.246736	-0.706406
Cl	5.277088	5.488679	-1.882350
Mg	6.322828	3.376073	-1.151917
Cl	8.443557	3.650014	-2.254952
Cl	1.904324	3.401289	-1.767362
Cl	-1.733483	-0.204431	1.871493
Mg	0.053664	-0.359983	0.098077
Cl	1.824002	-0.333520	-1.687050
Cl	-1.777008	3.523365	1.742057
Mg	0.067580	3.303305	-0.002074
Cl	-1.244193	1.509105	-1.106353
Cl	1.364206	1.548048	1.195790
Mg	-3.109899	1.563321	0.669625
Cl	-4.292368	-0.282967	-0.427019
Cl	-4.986469	1.739191	2.323885
Cl	-4.274995	3.444442	-0.688621
Mg	3.216462	1.485156	-0.606556
Cl	4.422487	3.427236	0.635562
Cl	5.086948	1.558818	-2.289525
Cl	4.389838	-0.294533	0.583542
Cl	7.455688	1.538105	0.303108
Cl	-7.303586	1.582024	-0.309739
C	-6.249110	-1.174871	-3.571597
C	-6.257153	-1.980522	-2.422920
C	-5.162177	-1.206882	-4.447801
H	-5.153637	-0.555072	-5.322723
C	-5.165554	-2.842212	-2.173342
C	-4.074833	-2.044824	-4.185627
H	-3.210823	-2.052274	-4.852047

C	-4.078394	-2.864359	-3.052984
H	-3.227282	-3.512890	-2.839288
H	-7.077763	-0.485975	-3.736760
C	-7.298116	-1.754735	-1.389074
C	-5.079565	-3.743405	-0.979761
O	-7.041217	-1.828280	-0.170189
O	-4.257002	-3.552308	-0.080877
O	-8.484601	-1.435788	-1.873599
C	-9.553924	-1.111274	-0.893198
H	-9.079888	-0.652320	-0.020228
H	-10.178577	-0.369947	-1.404392
C	-10.317108	-2.365512	-0.484941
H	-9.569575	-3.086309	-0.110030
O	-5.823219	-4.840111	-0.901335
C	-6.619666	-5.329285	-2.038051
H	-6.278926	-4.842873	-2.964028
H	-6.371994	-6.400649	-2.080539
C	-8.112820	-5.126309	-1.810481
H	-8.309689	-4.042024	-1.829932
C	6.551522	-0.896322	3.642767
C	6.508444	-1.809491	2.582278
C	5.476485	-0.806050	4.531766
H	5.503527	-0.073116	5.339595
C	5.385927	-2.652222	2.427480
C	4.357134	-1.626041	4.365165
H	3.504985	-1.538141	5.040922
C	4.311741	-2.550477	3.316790
H	3.433147	-3.180404	3.171925
H	7.404538	-0.222092	3.725960
C	7.521515	-1.715632	1.501523
C	5.273883	-3.642167	1.313406

O	7.192290	-1.756746	0.302168
O	4.342242	-3.606989	0.501490
O	8.762961	-1.570712	1.921119
C	9.818591	-1.296897	0.906334
H	9.341272	-0.836903	0.032703
H	10.466360	-0.562372	1.397051
O	6.149038	-4.634893	1.200471
C	7.149470	-4.905662	2.249390
H	7.921460	-4.124014	2.215279
H	6.653502	-4.874324	3.233245
C	7.749462	-6.274622	1.958820
H	8.124725	-6.245959	0.920927
C	10.562456	-2.566558	0.505491
H	10.893836	-3.067436	1.433633
C	6.697606	-7.384777	2.078974
H	5.846614	-7.202924	1.407264
H	7.132528	-8.361494	1.821221
H	6.316728	-7.448812	3.112087
C	8.940413	-6.500723	2.903405
H	8.619126	-6.495977	3.957983
H	9.406207	-7.476340	2.703799
H	9.712695	-5.725910	2.777485
C	-8.872159	-5.767311	-2.982634
H	-8.566380	-5.343216	-3.951897
H	-8.701194	-6.855651	-3.013854
H	-9.952532	-5.602042	-2.869925
C	-8.565689	-5.683966	-0.456445
H	-9.645475	-5.525811	-0.320331
H	-8.373291	-6.767516	-0.395494
H	-8.038803	-5.197240	0.376281
C	-11.259974	-2.012419	0.676927

H	-11.825988	-2.903530	0.987204
H	-10.695799	-1.639755	1.544557
H	-11.971292	-1.226586	0.383375
C	-11.069015	-2.977897	-1.673282
H	-10.403171	-3.159170	-2.530400
H	-11.530455	-3.936141	-1.390479
H	-11.873703	-2.303820	-2.007648
C	9.680862	-3.525554	-0.309403
H	10.253194	-4.421315	-0.593408
H	8.790467	-3.859606	0.241001
H	9.329087	-3.032721	-1.228580
C	11.803185	-2.150187	-0.305426
H	12.462199	-1.479814	0.263541
H	12.375215	-3.042033	-0.601388
H	11.505357	-1.612362	-1.218222
C	-2.624439	-6.374951	-0.487217
H	-3.395122	-6.129586	-1.234418
H	-2.603094	-7.468212	-0.363841
H	-2.916643	-5.913367	0.464838
C	-0.861630	-6.415897	-2.333200
H	0.114521	-6.028881	-2.655196
H	-0.808921	-7.514804	-2.303233
H	-1.613720	-6.128802	-3.085140

124

II_re_DEP SCF Done: -20313.5502564 □.U.

Mg	-3.232042	-2.539830	0.624366
Cl	-5.071369	-2.379985	2.215189
Mg	-6.393187	-0.746154	1.020394
Cl	-8.395239	-0.470072	2.381531
Mg	-9.360043	1.463504	1.300616
Cl	-11.423720	1.188819	0.408915

Ti	0.012867	-4.519010	0.109132
Cl	1.710153	-4.371354	-1.568029
C	0.859494	-5.955957	1.413774
H	1.574830	-6.466519	0.739236
C	1.582946	-5.418198	2.649087
H	0.092720	-6.695362	1.702316
H	0.928809	-4.768265	3.250762
H	1.944480	-6.230411	3.304950
H	2.455722	-4.826724	2.339292
Cl	-1.804819	-4.311769	1.680527
Mg	3.181633	-2.585004	-0.534012
Cl	5.003080	-2.508061	-2.137942
Mg	6.329990	-0.829292	-1.010415
Cl	8.336652	-0.646665	-2.383092
Mg	9.315570	1.304651	-1.364787
Cl	8.357121	3.304472	-2.325608
Mg	6.243130	3.088308	-1.187894
Cl	5.195066	5.195460	-1.919302
Mg	3.140379	4.963191	-0.719682
Cl	1.969710	7.096606	-1.290033
Mg	0.011385	6.568588	-0.067471
Cl	1.282498	4.883648	1.191917
Cl	1.227917	-2.502274	1.196105
Cl	-1.357575	-2.438083	-1.160533
Cl	11.411694	1.147280	-0.518679
Cl	-1.276440	4.880850	-1.301359
Cl	-1.940302	7.144601	1.141126
Mg	-3.142037	5.017331	0.598675
Cl	-5.202407	5.304493	1.773062
Mg	-6.266527	3.186114	1.087871
Cl	-8.392152	3.483339	2.194558

Cl	-1.856786	3.191901	1.721503
Cl	1.805180	-0.653925	-1.722921
Mg	-0.048951	-0.673440	-0.010421
Cl	-1.857371	-0.565450	1.747637
Cl	1.832570	3.123203	-1.795576
Mg	-0.013965	3.012723	-0.031295
Cl	1.261018	1.237811	1.142446
Cl	-1.310717	1.248487	-1.181899
Mg	3.129654	1.205845	-0.628838
Cl	4.301746	-0.520547	0.642394
Cl	5.027252	1.230971	-2.274903
Cl	4.340676	3.154347	0.613416
Mg	-3.180489	1.255882	0.595258
Cl	-4.354450	3.190247	-0.700851
Cl	-5.071554	1.358045	2.238056
Cl	-4.368776	-0.501244	-0.624304
Cl	-7.416966	1.395263	-0.381204
Cl	7.388553	1.324657	0.327403
C	6.317280	-0.911712	3.787133
C	6.344701	-1.871125	2.765338
C	5.207300	-0.812904	4.629078
H	5.181083	-0.044092	5.402810
C	5.255618	-2.756550	2.602808
C	4.120527	-1.672690	4.453222
H	3.237507	-1.582393	5.087824
C	4.144634	-2.641325	3.445467
H	3.286935	-3.297428	3.296885
H	7.147377	-0.210173	3.872762
C	7.405885	-1.781330	1.733602
C	5.175610	-3.796213	1.525620
O	7.157053	-1.953848	0.523450

O	4.232297	-3.804527	0.723593
O	8.597563	-1.490665	2.214674
C	9.697070	-1.137501	1.272595
H	9.394483	-1.416551	0.254133
H	9.782090	-0.045686	1.338942
C	10.963170	-1.829574	1.718156
H	11.779049	-1.470051	1.074323
H	11.208360	-1.580488	2.760081
H	10.884481	-2.921313	1.616772
O	6.043503	-4.794088	1.436520
C	7.166039	-5.002599	2.364507
H	7.181261	-4.214325	3.129204
H	6.951048	-5.963306	2.853656
C	8.447692	-5.054129	1.553952
H	9.298965	-5.227094	2.228854
H	8.410812	-5.868632	0.818181
H	8.611975	-4.112004	1.014596
C	-6.302524	-1.067097	-3.780684
C	-6.365072	-1.968372	-2.709750
C	-5.175576	-1.035994	-4.606227
H	-5.121216	-0.310976	-5.419782
C	-5.294218	-2.860672	-2.481795
C	-4.106762	-1.903686	-4.366274
H	-3.211485	-1.860198	-4.988419
C	-4.165856	-2.814483	-3.307620
H	-3.323223	-3.474043	-3.098251
H	-7.116295	-0.354887	-3.919647
C	-7.437820	-1.819194	-1.697489
C	-5.255409	-3.839687	-1.351971
O	-7.211106	-1.949335	-0.477946
O	-4.314743	-3.843268	-0.546687

O -8.621337 -1.522368 -2.197921

C -9.712963 -1.221931 -1.230047

H -9.259109 -0.757624 -0.350058

H -10.332449 -0.484341 -1.748853

O -6.165463 -4.797752 -1.226452

C -7.252728 -4.981420 -2.202507

H -8.072228 -4.306701 -1.913715

H -6.894073 -4.701492 -3.204046

C -7.675884 -6.433414 -2.124726

H -8.513315 -6.605308 -2.817034

H -6.848403 -7.099129 -2.406496

H -8.005426 -6.689390 -1.108638

C -10.459437 -2.481428 -0.851447

H -10.875915 -2.986141 -1.735028

H -9.807528 -3.173190 -0.298521

H -11.288673 -2.192220 -0.189366

C -1.495965 -5.620668 -1.449698

H -2.436950 -5.320319 -0.983447

H -1.237622 -5.145567 -2.401360

C -0.801741 -6.717005 -1.013910

H -1.161287 -7.222809 -0.111024

C 0.294609 -7.404292 -1.768137

H 1.157074 -7.628033 -1.122606

H -0.082442 -8.371610 -2.144656

H 0.641478 -6.808542 -2.622449

124

II_si_DEP SCF Done: -20313.5461813 ⍰.U.

Mg 3.187237 -2.522096 -0.651170

Cl 5.035079 -2.347882 -2.226222

Mg 6.332083 -0.678764 -1.055259

Cl 8.314754 -0.379685 -2.444296

Mg	9.281329	1.555342	-1.366800
Cl	11.367108	1.304771	-0.520903
Ti	0.012317	-4.523352	-0.074669
Cl	-1.722906	-4.385275	1.643650
C	-0.513007	-6.503568	-0.593152
H	-0.986909	-7.061339	0.222580
C	-1.490339	-5.839798	-1.536076
H	0.262480	-7.093216	-1.095245
H	-1.295488	-4.733062	-1.665835
H	-1.396844	-6.195125	-2.572309
H	-2.530985	-5.886491	-1.189870
Cl	1.785487	-4.284755	-1.721220
Mg	-3.177844	-2.642307	0.663428
Cl	-5.062128	-2.505054	2.198101
Mg	-6.373738	-0.882557	0.979965
Cl	-8.432823	-0.709479	2.277428
Mg	-9.390670	1.223431	1.205272
Cl	-8.488200	3.241707	2.186078
Mg	-6.333537	3.041592	1.124114
Cl	-5.323146	5.163272	1.873503
Mg	-3.240597	4.943316	0.721899
Cl	-2.087865	7.082418	1.322978
Mg	-0.119155	6.583026	0.106398
Cl	-1.366489	4.896185	-1.171417
Cl	-1.260176	-2.443253	-1.143199
Cl	1.296206	-2.445765	1.148576
Cl	-11.445372	1.035263	0.268872
Cl	1.188765	4.899636	1.329330
Cl	1.828474	7.183146	-1.099000
Mg	3.045346	5.059598	-0.578550
Cl	5.093015	5.365178	-1.770674

Mg	6.178299	3.251878	-1.103601
Cl	8.287132	3.573147	-2.233621
Cl	1.771694	3.226628	-1.701439
Cl	-1.842579	-0.626631	1.775768
Mg	-0.002504	-0.636778	0.040084
Cl	1.800787	-0.534556	-1.735494
Cl	-1.921430	3.123706	1.811416
Mg	-0.066851	3.030888	0.060406
Cl	-1.319057	1.250719	-1.120501
Cl	1.258684	1.272662	1.201800
Mg	-3.198734	1.176710	0.637499
Cl	-4.317158	-0.600084	-0.619039
Cl	-5.119401	1.212103	2.253836
Cl	-4.397571	3.116949	-0.630778
Mg	3.112329	1.297174	-0.598952
Cl	4.285796	3.233808	0.701074
Cl	4.993255	1.416143	-2.254318
Cl	4.319904	-0.451545	0.603008
Cl	7.364435	1.461351	0.344680
Cl	-7.407294	1.247539	-0.409892
C	-6.145981	-1.207118	-3.864804
C	-6.235828	-2.084858	-2.775622
C	-5.007468	-1.205388	-4.673997
H	-4.932608	-0.497986	-5.501285
C	-5.181478	-2.987923	-2.514782
C	-3.953813	-2.081743	-4.401061
H	-3.049028	-2.063070	-5.010604
C	-4.041763	-2.971509	-3.326932
H	-3.212714	-3.641399	-3.096873
H	-6.951388	-0.492067	-4.033916
C	-7.333049	-1.907721	-1.793047

C	-5.153210	-3.945377	-1.363899
O	-7.140180	-2.036824	-0.567961
O	-4.210150	-3.924404	-0.562207
O	-8.494121	-1.604694	-2.337152
C	-9.631737	-1.209608	-1.458644
H	-9.378587	-1.457809	-0.418968
H	-9.701813	-0.120064	-1.563320
C	-10.881438	-1.908473	-1.938630
H	-11.721701	-1.527785	-1.339987
H	-11.080447	-1.688929	-2.996896
H	-10.811765	-2.997066	-1.802546
O	-6.051334	-4.908009	-1.206699
C	-7.186616	-5.142949	-2.112401
H	-7.192011	-4.399543	-2.921007
H	-6.998167	-6.134036	-2.549526
C	-8.461366	-5.120743	-1.289729
H	-9.321715	-5.316857	-1.946653
H	-8.433666	-5.891990	-0.508408
H	-8.600805	-4.145734	-0.804582
C	6.359568	-0.894846	3.720162
C	6.391255	-1.829774	2.677264
C	5.250358	-0.826140	4.566987
H	5.219294	-0.075550	5.358223
C	5.306798	-2.716503	2.495572
C	4.169864	-1.691204	4.376145
H	3.288744	-1.622226	5.015935
C	4.197239	-2.632937	3.343468
H	3.343058	-3.290004	3.180601
H	7.182739	-0.186677	3.818218
C	7.444433	-1.719941	1.640096
C	5.240993	-3.722972	1.390923

O 7.189858 -1.875653 0.429011

O 4.294325 -3.738050 0.592737

O 8.641828 -1.427098 2.108457

C 9.714614 -1.158237 1.111176

H 9.245823 -0.713488 0.228339

H 10.349443 -0.412271 1.598520

O 6.136713 -4.697101 1.281453

C 7.233457 -4.870144 2.249232

H 8.060218 -4.219397 1.928014

H 6.893262 -4.552185 3.245702

C 7.628329 -6.331593 2.212392

H 8.473045 -6.497061 2.897307

H 6.792997 -6.971366 2.528972

H 7.937243 -6.626857 1.200463

C 10.444250 -2.433097 0.750949

H 10.871440 -2.919101 1.639912

H 9.777215 -3.132982 0.226988

H 11.265104 -2.169293 0.068196

C 1.628023 -5.458042 1.891101

H 2.409269 -4.701161 1.834535

H 0.835191 -5.318293 2.628869

C 1.751254 -6.590102 1.162447

H 1.005286 -7.378924 1.291616

C 2.902353 -6.898615 0.257696

H 3.589861 -6.048412 0.161775

H 3.453928 -7.768866 0.653258

H 2.555699 -7.173994 -0.749822

148

II_RE_DIBP SCF Done: -20628.1878282 ⍰.U.

Mg -3.216910 -2.223169 0.613488

Cl -4.986402 -2.091125 2.288934

Mg	-6.335806	-0.422752	1.179932
Cl	-8.341828	-0.160007	2.564520
Mg	-9.302917	1.770374	1.459241
Cl	-11.384055	1.632530	0.586727
Ti	0.017163	-4.222663	0.028831
Cl	1.678831	-4.089155	-1.696956
C	0.889670	-5.653033	1.318543
H	1.530066	-6.232216	0.623494
C	1.707473	-5.117267	2.494355
H	0.096647	-6.326769	1.680342
H	1.108733	-4.440127	3.123117
H	2.083809	-5.930204	3.141210
H	2.576322	-4.556284	2.124726
Cl	-1.782225	-4.022975	1.617082
Mg	3.173626	-2.279812	-0.736077
Cl	4.933718	-2.185705	-2.402536
Mg	6.314546	-0.588500	-1.226770
Cl	8.287767	-0.323832	-2.649040
Mg	9.304421	1.580900	-1.546608
Cl	8.345192	3.614642	-2.425712
Mg	6.237880	3.354479	-1.293654
Cl	5.199347	5.494044	-1.967068
Mg	3.168637	5.258891	-0.734670
Cl	1.995414	7.409908	-1.237094
Mg	0.061145	6.870006	0.017767
Cl	1.350581	5.152048	1.214014
Cl	1.264236	-2.218480	1.074033
Cl	-1.382747	-2.095811	-1.200060
Cl	11.397434	1.407578	-0.706691
Cl	-1.267391	5.215376	-1.220270
Cl	-1.859692	7.430624	1.282436

Mg	-3.087701	5.322717	0.719668
Cl	-5.124767	5.594578	1.934592
Mg	-6.212267	3.491380	1.226374
Cl	-8.310349	3.775874	2.371861
Cl	-1.786551	3.472642	1.782888
Cl	1.763384	-0.330886	-1.835193
Mg	-0.036817	-0.363693	-0.061433
Cl	-1.794786	-0.277267	1.750130
Cl	1.820430	3.455327	-1.823608
Mg	0.017423	3.316187	-0.016889
Cl	1.314478	1.510695	1.084341
Cl	-1.317347	1.583870	-1.168025
Mg	3.134195	1.501441	-0.746690
Cl	4.321563	-0.257517	0.457871
Cl	4.991698	1.554896	-2.426915
Cl	4.380141	3.406055	0.535746
Mg	-3.138155	1.561963	0.658240
Cl	-4.339666	3.516222	-0.585673
Cl	-5.000728	1.649913	2.348902
Cl	-4.366057	-0.167300	-0.550251
Cl	-7.396412	1.685291	-0.229732
Cl	7.388855	1.509061	0.152121
C	6.436040	-0.679124	3.506671
C	6.372942	-1.674191	2.519996
C	5.367301	-0.489195	4.384839
H	5.413265	0.306984	5.129397
C	5.228283	-2.498919	2.432258
C	4.228721	-1.292062	4.282154
H	3.377882	-1.129389	4.945741
C	4.159278	-2.295925	3.310920
H	3.263711	-2.911129	3.220417

H	7.303586	-0.019401	3.532195
C	7.388273	-1.661040	1.439719
C	5.074098	-3.579095	1.401203
O	7.079497	-1.862229	0.247163
O	4.251188	-3.500412	0.483841
O	8.610816	-1.364313	1.842105
C	9.641666	-1.185832	0.787656
H	9.142986	-0.790444	-0.103155
H	10.319984	-0.427333	1.194620
C	10.338164	-2.503053	0.468151
H	9.549708	-3.235260	0.219803
O	5.767917	-4.706261	1.495346
C	6.576584	-5.033720	2.680146
H	6.279357	-4.387287	3.519113
H	6.291348	-6.072011	2.906275
C	8.069494	-4.931547	2.387411
H	8.305137	-3.869400	2.212848
C	-6.527523	-0.651689	-3.574961
C	-6.496229	-1.609266	-2.554328
C	-5.443961	-0.526184	-4.449605
H	-5.461458	0.242126	-5.224128
C	-5.377172	-2.461659	-2.427179
C	-4.326836	-1.353505	-4.307230
H	-3.466252	-1.234675	-4.967271
C	-4.292699	-2.321706	-3.298934
H	-3.413276	-2.953132	-3.165629
H	-7.376157	0.030536	-3.633942
C	-7.507295	-1.541773	-1.469075
C	-5.270428	-3.495479	-1.355184
O	-7.174829	-1.621030	-0.272747
O	-4.328332	-3.507563	-0.552370

O	-8.748393	-1.369635	-1.879995
C	-9.793213	-1.101684	-0.851801
H	-9.302133	-0.669134	0.028261
H	-10.431529	-0.345410	-1.321435
O	-6.155884	-4.481753	-1.272484
C	-7.163634	-4.706180	-2.325356
H	-7.933223	-3.924787	-2.254101
H	-6.672827	-4.635171	-3.309718
C	-7.764804	-6.084823	-2.087824
H	-8.137136	-6.097141	-1.048569
C	-10.556287	-2.366982	-0.474472
H	-10.915383	-2.833518	-1.410187
C	-6.714775	-7.190704	-2.255330
H	-5.862467	-7.036961	-1.578285
H	-7.150496	-8.176535	-2.036629
H	-6.335876	-7.212919	-3.290885
C	-8.958069	-6.272241	-3.038013
H	-8.638143	-6.228843	-4.092112
H	-9.426403	-7.253339	-2.874352
H	-9.728060	-5.500675	-2.882076
C	8.841306	-5.388045	3.635256
H	8.587850	-4.782541	4.519439
H	8.625593	-6.443564	3.867835
H	9.923370	-5.298650	3.465248
C	8.464243	-5.731882	1.141016
H	9.543952	-5.638198	0.954441
H	8.235259	-6.801829	1.274424
H	7.928817	-5.378033	0.248754
C	11.212605	-2.300503	-0.780014
H	11.738710	-3.234258	-1.029118
H	10.602738	-2.003590	-1.646062

H	11.959385	-1.509399	-0.614672
C	11.149282	-3.012734	1.666252
H	10.532509	-3.089988	2.574278
H	11.574606	-4.005884	1.456436
H	11.984925	-2.328345	1.882772
C	-9.681023	-3.368114	0.295311
H	-10.268253	-4.258361	0.566114
H	-8.810162	-3.707062	-0.283037
H	-9.298768	-2.909922	1.220201
C	-11.772954	-1.949246	0.371555
H	-12.425332	-1.245075	-0.163119
H	-12.360821	-2.837035	0.648092
H	-11.446809	-1.449107	1.295973
C	-1.493753	-5.262282	-1.540274
H	-2.488458	-5.059761	-1.133291
H	-1.169620	-4.647940	-2.384492
C	-0.799147	-6.390758	-1.196812
H	0.130309	-6.614614	-1.731947
C	-1.325326	-7.446823	-0.272826
H	-1.799492	-8.241488	-0.876229
H	-0.525514	-7.921615	0.310941
H	-2.082455	-7.044013	0.415205

148

II_SI_DIBP SCF Done: -20628.1882742 ?.U.

Mg	-3.210577	-2.232576	0.648566
Cl	-4.973917	-2.087638	2.327810
Mg	-6.335510	-0.438801	1.204036
Cl	-8.336640	-0.169238	2.594490
Mg	-9.312459	1.742573	1.470042
Cl	-11.397550	1.583276	0.610482
Ti	0.027545	-4.205948	0.033064

Cl	1.661127	-4.041700	-1.707585
C	0.928469	-5.630228	1.314957
H	1.660221	-6.096600	0.625340
C	1.633263	-5.099340	2.563831
H	0.191547	-6.405565	1.586776
H	0.955605	-4.489626	3.181510
H	2.025244	-5.914017	3.198763
H	2.482367	-4.466429	2.272133
Cl	-1.751072	-4.017122	1.649546
Mg	3.169565	-2.264099	-0.713204
Cl	4.937449	-2.190236	-2.370629
Mg	6.307905	-0.579176	-1.201913
Cl	8.287889	-0.321440	-2.615114
Mg	9.295845	1.590075	-1.515630
Cl	8.340842	3.618623	-2.413221
Mg	6.223508	3.362909	-1.299617
Cl	5.181258	5.493013	-2.000916
Mg	3.144366	5.265676	-0.776601
Cl	1.962116	7.405323	-1.304923
Mg	0.027155	6.869954	-0.048420
Cl	1.321083	5.170009	1.167477
Cl	1.263315	-2.197898	1.088000
Cl	-1.387440	-2.125840	-1.189667
Cl	11.383100	1.418671	-0.661291
Cl	-1.295807	5.197611	-1.269577
Cl	-1.896554	7.436242	1.209611
Mg	-3.116160	5.317203	0.671178
Cl	-5.150921	5.592176	1.888855
Mg	-6.231268	3.476984	1.205428
Cl	-8.327121	3.764937	2.353646
Cl	-1.806464	3.482170	1.749842

Cl	1.757470	-0.328114	-1.837651
Mg	-0.046793	-0.365907	-0.070421
Cl	-1.795336	-0.270097	1.748955
Cl	1.803622	3.449721	-1.853688
Mg	-0.003263	3.316116	-0.049487
Cl	1.296298	1.526425	1.068729
Cl	-1.334471	1.567661	-1.185614
Mg	3.120131	1.510946	-0.755568
Cl	4.306471	-0.235915	0.467828
Cl	4.987073	1.552942	-2.426622
Cl	4.356319	3.427600	0.517039
Mg	-3.151188	1.555894	0.646979
Cl	-4.364231	3.492410	-0.613337
Cl	-5.007087	1.654040	2.343979
Cl	-4.373649	-0.192145	-0.536951
Cl	-7.412180	1.647842	-0.225515
Cl	7.368840	1.526692	0.168717
C	6.400424	-0.629305	3.528339
C	6.338931	-1.635995	2.553286
C	5.328508	-0.425289	4.399363
H	5.373597	0.379890	5.134228
C	5.192271	-2.458786	2.470208
C	4.188365	-1.226295	4.301565
H	3.335024	-1.053618	4.959380
C	4.120786	-2.241950	3.342568
H	3.223815	-2.855867	3.257412
H	7.269851	0.028059	3.549900
C	7.360098	-1.636109	1.478381
C	5.040118	-3.553554	1.453581
O	7.057557	-1.846572	0.285893
O	4.224462	-3.486058	0.528735

O	8.581127	-1.338211	1.884849
C	9.617388	-1.168769	0.834334
H	9.123977	-0.778115	-0.061599
H	10.295060	-0.408720	1.239549
C	10.313617	-2.489150	0.527450
H	9.525626	-3.222475	0.280711
O	5.730214	-4.681194	1.568990
C	6.533097	-4.991643	2.762348
H	6.232058	-4.332913	3.590312
H	6.246538	-6.026459	3.002227
C	8.027526	-4.893874	2.475372
H	8.264089	-3.834614	2.285251
C	-6.552228	-0.711221	-3.544131
C	-6.509626	-1.660533	-2.516213
C	-5.473892	-0.585662	-4.425157
H	-5.500264	0.176265	-5.205715
C	-5.384299	-2.504311	-2.387550
C	-4.350618	-1.404407	-4.281544
H	-3.493926	-1.285001	-4.946540
C	-4.305127	-2.364245	-3.265722
H	-3.420041	-2.987166	-3.130443
H	-7.405514	-0.034916	-3.604220
C	-7.515491	-1.590478	-1.426365
C	-5.266424	-3.527684	-1.306579
O	-7.175987	-1.655995	-0.231147
O	-4.323061	-3.524217	-0.505266
O	-8.759840	-1.431153	-1.832376
C	-9.801178	-1.159997	-0.801607
H	-9.308258	-0.717835	0.072641
H	-10.445629	-0.410914	-1.274391
O	-6.143916	-4.519797	-1.213313

C	-7.152416	-4.760297	-2.262065
H	-7.928385	-3.984926	-2.193949
H	-6.664835	-4.691677	-3.248192
C	-7.740850	-6.142542	-2.013964
H	-8.110295	-6.151430	-0.973645
C	-10.555770	-2.425867	-0.409962
H	-10.920050	-2.900545	-1.339527
C	-6.681549	-7.240147	-2.177398
H	-5.828310	-7.074884	-1.504262
H	-7.107907	-8.228337	-1.951023
H	-6.305863	-7.265756	-3.214066
C	-8.934941	-6.346323	-2.959748
H	-8.618197	-6.306866	-4.014979
H	-9.394059	-7.330491	-2.788629
H	-9.711273	-5.580579	-2.806637
C	8.792967	-5.330693	3.734165
H	8.535480	-4.710877	4.607179
H	8.575410	-6.382191	3.982633
H	9.875960	-5.244754	3.568271
C	8.428780	-5.713382	1.243617
H	9.509086	-5.620735	1.060056
H	8.201072	-6.781456	1.393240
H	7.896606	-5.374650	0.343580
C	11.194429	-2.296007	-0.717732
H	11.722663	-3.231250	-0.956518
H	10.589020	-2.006518	-1.589376
H	11.939783	-1.503041	-0.554660
C	11.118478	-2.990966	1.733085
H	10.496998	-3.061941	2.638386
H	11.544591	-3.985601	1.532140
H	11.953173	-2.305324	1.949318

C -9.670334 -3.417804 0.360028

H -10.251485 -4.308878 0.640992

H -8.802223 -3.756928 -0.222444

H -9.283487 -2.951856 1.279092

C -11.767606 -2.007049 0.442557

H -12.426842 -1.309084 -0.091818

H -12.349792 -2.895185 0.729778

H -11.436273 -1.499391 1.361045

C -1.521303 -5.312690 -1.486419

H -2.449784 -5.012003 -0.996102

H -1.289388 -4.839671 -2.445696

C -0.812980 -6.404312 -1.062146

H -1.150097 -6.905974 -0.148217

C 0.266898 -7.094241 -1.837360

H 0.590866 -6.504935 -2.704990

H 1.145273 -7.309677 -1.210724

H -0.115811 -8.065953 -2.196493

124

TS_RE_DEP SCF Done: -20313.5328633 ⍰.U.

Mg -3.192796 -2.518706 0.736231

Cl -5.032829 -2.297905 2.318976

Mg -6.350453 -0.691379 1.081591

Cl -8.346671 -0.379513 2.446326

Mg -9.316459 1.528838 1.325743

Cl -11.390895 1.242281 0.461827

Ti -0.111157 -4.501816 -0.011602

Cl 1.764791 -4.390275 -1.594884

C 0.631597 -6.653200 0.417053

H 1.424190 -6.864445 -0.307634

C 1.155381 -5.854085 1.602600

H 0.191987 -7.581551 0.788971

H	2.224837	-6.079174	1.741126
H	1.212648	-4.738337	1.505438
H	0.592640	-6.037229	2.525240
Cl	-1.781031	-4.322926	1.742726
Mg	3.155553	-2.602989	-0.602916
Cl	5.052314	-2.493771	-2.126935
Mg	6.349307	-0.838088	-0.940724
Cl	8.411411	-0.676416	-2.236234
Mg	9.357603	1.277206	-1.191350
Cl	8.447600	3.276855	-2.202875
Mg	6.290437	3.081508	-1.144595
Cl	5.268910	5.184567	-1.932374
Mg	3.185145	4.971966	-0.780404
Cl	2.018809	7.093042	-1.416525
Mg	0.051255	6.598736	-0.194877
Cl	1.309298	4.942900	1.112323
Cl	1.238932	-2.412616	1.177221
Cl	-1.321791	-2.431081	-1.110005
Cl	11.412500	1.110738	-0.250735
Cl	-1.242378	4.886574	-1.391883
Cl	-1.902368	7.203713	0.998004
Mg	-3.103852	5.062290	0.509081
Cl	-5.157972	5.377867	1.687475
Mg	-6.225260	3.244799	1.055288
Cl	-8.344262	3.568941	2.167724
Cl	-1.819708	3.258859	1.666315
Cl	1.821220	-0.616074	-1.753662
Mg	-0.017471	-0.621500	-0.014144
Cl	-1.816560	-0.505729	1.766743
Cl	1.878298	3.127757	-1.841875
Mg	0.021900	3.048191	-0.088920

Cl	1.284993	1.295846	1.119117
Cl	-1.291313	1.263567	-1.201921
Mg	3.163623	1.208981	-0.637952
Cl	4.289410	-0.540110	0.649068
Cl	5.090180	1.227751	-2.249689
Cl	4.352120	3.174817	0.603224
Mg	-3.146019	1.297022	0.592601
Cl	-4.326243	3.206163	-0.742388
Cl	-5.028196	1.441175	2.242278
Cl	-4.336135	-0.491946	-0.574273
Cl	-7.385910	1.421712	-0.365214
Cl	7.372023	1.315570	0.419941
C	6.118225	-1.075188	3.899796
C	6.210325	-1.977312	2.830828
C	4.977375	-1.053188	4.705286
H	4.900938	-0.326710	5.515725
C	5.155203	-2.884340	2.586081
C	3.923271	-1.933654	4.448510
H	3.016178	-1.899568	5.053917
C	4.013509	-2.847413	3.394979
H	3.182349	-3.518510	3.176423
H	6.923797	-0.357090	4.054613
C	7.309480	-1.818265	1.847258
C	5.126103	-3.867499	1.456296
O	7.118505	-1.967628	0.624355
O	4.179913	-3.869044	0.658183
O	8.469840	-1.506006	2.387910
C	9.606442	-1.119971	1.504026
H	9.352087	-1.379244	0.467379
H	9.675967	-0.029307	1.597077
C	10.857346	-1.812792	1.989589

H	10.788507	-2.902785	1.864737
O	6.028724	-4.829729	1.316889
C	7.168207	-5.041051	2.222942
H	7.175000	-4.280015	3.014838
H	6.984894	-6.022699	2.683177
C	8.439735	-5.033941	1.394964
H	8.410941	-5.822394	0.631028
C	-6.284864	-1.104117	-3.714528
C	-6.346633	-1.980656	-2.623269
C	-5.163973	-1.101627	-4.548837
H	-5.109443	-0.395625	-5.378947
C	-5.281104	-2.875964	-2.383033
C	-4.101384	-1.974116	-4.298312
H	-3.211299	-1.953539	-4.929082
C	-4.159054	-2.859857	-3.218344
H	-3.321957	-3.525065	-3.004077
H	-7.093959	-0.388700	-3.863848
C	-7.413357	-1.806488	-1.608763
C	-5.244329	-3.826378	-1.229252
O	-7.182970	-1.921693	-0.388196
O	-4.306213	-3.811230	-0.422230
O	-8.596481	-1.507929	-2.109344
C	-9.684930	-1.188780	-1.144515
H	-9.225209	-0.745336	-0.256659
H	-10.284168	-0.431755	-1.659174
O	-6.155732	-4.781191	-1.083705
C	-7.241499	-4.982284	-2.057402
H	-8.057702	-4.295151	-1.789120
H	-6.878286	-4.730624	-3.064824
C	-1.471441	-5.435763	-1.420655
H	-2.446425	-5.069196	-1.090228

H -1.143073 -5.116443 -2.415555

C -1.023861 -6.688612 -0.926939

H 11.057548 -1.582171 3.045280

H 11.696516 -1.437543 1.385985

H 9.303159 -5.213288 2.052652

H 8.574713 -4.069606 0.887757

C -10.463219 -2.434169 -0.783517

H -10.886155 -2.919833 -1.674611

H -9.830574 -3.147151 -0.235011

H -11.289159 -2.132515 -0.122880

C -7.674693 -6.428993 -1.943589

H -8.511592 -6.613233 -2.633346

H -6.850916 -7.107110 -2.206046

H -8.008244 -6.656645 -0.922057

C -0.347865 -7.647494 -1.881669

H 0.121017 -8.505427 -1.380407

H -1.115371 -8.037619 -2.570133

H 0.411246 -7.125993 -2.483451

H -1.672254 -7.144802 -0.174126

130

TS_si_dep SCF Done: -20392.1953623 [illegible].U.

Mg -3.170757 -2.484783 0.763745

Cl -5.027449 -2.267132 2.334006

Mg -6.343927 -0.673712 1.085294

Cl -8.370846 -0.427865 2.423290

Mg -9.331813 1.487586 1.325406

Cl -11.420232 1.303937 0.462562

Ti 0.117851 -4.493816 0.184494

Cl 1.774243 -4.234897 -1.575443

C -0.580615 -6.675677 -0.104361

H -1.339263 -6.785402 0.680327

C	-1.135778	-6.040155	-1.375444
H	-0.166798	-7.653175	-0.361870
H	-0.997815	-4.917865	-1.429929
Cl	-1.793901	-4.301094	1.738717
Mg	3.194175	-2.460828	-0.567126
Cl	5.053548	-2.339521	-2.145109
Mg	6.359158	-0.676593	-0.979919
Cl	8.395677	-0.485553	-2.310949
Mg	9.336582	1.476720	-1.278124
Cl	8.380723	3.464408	-2.268171
Mg	6.248776	3.238159	-1.163149
Cl	5.191219	5.330843	-1.920029
Mg	3.125569	5.082535	-0.741858
Cl	1.935373	7.194250	-1.358675
Mg	-0.018755	6.662033	-0.133707
Cl	1.263798	5.014302	1.158820
Cl	1.285446	-2.339067	1.234953
Cl	-1.274823	-2.462722	-1.060062
Cl	11.410815	1.332851	-0.376414
Cl	-1.291938	4.939237	-1.335877
Cl	-1.974800	7.243500	1.064863
Mg	-3.152647	5.093228	0.567855
Cl	-5.208969	5.389757	1.749882
Mg	-6.259303	3.256017	1.104166
Cl	-8.380171	3.521290	2.221967
Cl	-1.848247	3.293603	1.713998
Mg	-0.004692	-0.590459	0.030020
Cl	1.821637	-0.519712	-1.718335
Cl	-1.813200	-0.477524	1.793114
Cl	1.838660	3.223593	-1.804724
Mg	-0.008089	3.103322	-0.045437

Cl	1.280573	1.368025	1.156841
Cl	-1.294602	1.298526	-1.158544
Mg	3.148457	1.324565	-0.607694
Cl	4.322090	-0.409087	0.660263
Cl	5.053012	1.370193	-2.250398
Cl	4.333259	3.297432	0.619495
Mg	-3.154867	1.331002	0.629241
Cl	-4.353323	3.240842	-0.694697
Cl	-5.049118	1.449888	2.272426
Cl	-4.321341	-0.448475	-0.557637
Cl	-7.396605	1.442522	-0.346591
Cl	7.382436	1.487327	0.372956
C	6.218863	-0.948373	3.869216
C	6.309052	-1.836504	2.788707
C	5.091383	-0.957362	4.693793
H	5.014929	-0.241698	5.513809
C	5.267223	-2.760716	2.551982
C	4.050030	-1.855055	4.444807
H	3.153039	-1.845217	5.065939
C	4.138383	-2.755388	3.379347
H	3.317068	-3.441390	3.170042
H	7.013725	-0.217397	4.019125
C	7.387741	-1.650344	1.787886
C	5.242116	-3.731773	1.412753
O	7.177801	-1.794942	0.567288
O	4.289717	-3.737060	0.620217
O	8.552114	-1.323886	2.310666
C	9.668775	-0.918152	1.410144
H	9.403837	-1.182588	0.377384
H	9.720887	0.173623	1.501523
C	10.938185	-1.588887	1.878473

H	10.886171	-2.680074	1.755995
O	6.152462	-4.682542	1.257553
C	7.301444	-4.886486	2.153702
H	7.300427	-4.134364	2.954052
H	7.135782	-5.876059	2.603268
C	8.566910	-4.848924	1.317517
H	8.547481	-5.630622	0.546381
C	-6.257279	-1.032436	-3.738995
C	-6.329711	-1.904246	-2.643885
C	-5.129549	-1.034105	-4.563196
H	-5.066395	-0.331456	-5.395500
C	-5.271238	-2.804287	-2.392785
C	-4.069641	-1.905179	-4.297010
H	-3.171461	-1.885322	-4.916250
C	-4.141005	-2.789271	-3.216677
H	-3.302432	-3.447149	-2.986480
H	-7.065476	-0.318554	-3.899669
C	-7.405487	-1.721580	-1.639641
C	-5.236273	-3.749116	-1.231842
O	-7.184384	-1.841740	-0.417922
O	-4.314208	-3.698645	-0.411531
O	-8.578522	-1.422577	-2.159352
C	-9.692580	-1.012655	-1.258054
H	-9.418501	-1.256112	-0.222495
H	-9.756131	0.076722	-1.368921
O	-6.112323	-4.735691	-1.087101
C	-7.234207	-4.985084	-2.004430
H	-7.228579	-4.252653	-2.823138
H	-7.038474	-5.982016	-2.424405
C	1.512096	-5.333923	1.641293
H	2.477345	-4.893070	1.380180

H	1.078574	-5.032898	2.600285
C	1.205466	-6.607300	1.105162
H	11.149418	-1.353461	2.930950
H	11.762148	-1.200320	1.262487
H	9.437880	-5.018339	1.967802
H	8.680871	-3.877610	0.818550
C	-10.959132	-1.702701	-1.705741
H	-11.178262	-1.489451	-2.761342
H	-10.898110	-2.790931	-1.562420
H	-11.782846	-1.308454	-1.093004
C	-8.521392	-4.951705	-1.201411
H	-9.373051	-5.150580	-1.868733
H	-8.507866	-5.714857	-0.411771
H	-8.664882	-3.971528	-0.728041
C	-0.455527	-6.583035	-2.641390
H	0.637378	-6.514255	-2.570048
H	-0.734203	-7.639329	-2.781169
H	-0.772025	-6.021975	-3.532869
C	-2.668218	-6.201665	-1.424995
H	-3.082659	-5.712282	-2.321149
H	-2.923301	-7.271036	-1.474124
H	-3.143322	-5.765382	-0.537393
C	2.241229	-7.297608	0.247252
H	3.034581	-7.681763	0.910197
H	1.834310	-8.146988	-0.318172
H	2.704015	-6.593164	-0.456681
H	0.595923	-7.262796	1.731794

154

TS_re_DIBP SCF Done: -20706.8322519 ?.U.

Mg	3.219287	-2.139484	-0.850776
Cl	5.024273	-1.875753	-2.476666

Mg	6.354707	-0.282193	-1.240725
Cl	8.339310	0.061001	-2.632466
Mg	9.303362	1.966254	-1.493696
Cl	11.395405	1.761895	-0.655648
Ti	-0.019586	-4.212962	-0.189608
Cl	-1.630202	-4.006080	1.621627
C	0.727441	-6.386640	0.054208
H	1.466548	-6.472940	-0.751731
C	1.305079	-5.753680	1.316416
H	0.340285	-7.374926	0.312205
H	1.148781	-4.634632	1.384992
Cl	1.841021	-3.969132	-1.799921
Mg	-3.112885	-2.242420	0.684937
Cl	-4.908076	-2.173165	2.343220
Mg	-6.301230	-0.551675	1.219936
Cl	-8.339245	-0.348359	2.563332
Mg	-9.289977	1.591571	1.494888
Cl	-8.352568	3.600406	2.445251
Mg	-6.245058	3.376244	1.309409
Cl	-5.222329	5.498668	2.055630
Mg	-3.174197	5.292039	0.850799
Cl	-2.011900	7.424892	1.454780
Mg	-0.075493	6.932380	0.188904
Cl	-1.355825	5.262098	-1.081164
Cl	-1.256075	-2.072951	-1.176582
Cl	1.373568	-2.173145	1.035542
Cl	-11.351190	1.534479	0.553895
Cl	1.255478	5.229772	1.358649
Cl	1.841805	7.553390	-1.049440
Mg	3.070457	5.424243	-0.579513
Cl	5.096049	5.764050	-1.796448

Mg	6.196341	3.642254	-1.181072
Cl	8.291698	3.994318	-2.322444
Cl	1.772578	3.608488	-1.708399
Cl	-1.734139	-0.286778	1.808678
Mg	0.041098	-0.315518	0.003288
Cl	1.794616	-0.153774	-1.815799
Cl	-1.832024	3.459641	1.893852
Mg	-0.022706	3.372028	0.092202
Cl	-1.312767	1.616459	-1.077786
Cl	1.327357	1.589385	1.164550
Mg	-3.130847	1.529732	0.744296
Cl	-4.312767	-0.221717	-0.480992
Cl	-5.007556	1.551411	2.422088
Cl	-4.374537	3.469180	-0.486835
Mg	3.140263	1.664950	-0.672811
Cl	4.332616	3.581014	0.647070
Cl	4.991720	1.839379	-2.359784
Cl	4.366763	-0.107333	0.460592
Cl	7.397661	1.814550	0.209631
Cl	-7.377776	1.548667	-0.181017
C	-6.402698	-0.675723	-3.499843
C	-6.379062	-1.657417	-2.501373
C	-5.310841	-0.526789	-4.358975
H	-5.319708	0.261584	-5.113124
C	-5.260876	-2.511196	-2.379659
C	-4.196849	-1.360379	-4.227526
H	-3.330342	-1.227896	-4.877152
C	-4.171741	-2.352839	-3.243049
H	-3.293238	-2.987964	-3.122344
H	-7.253638	0.004218	-3.551086
C	-7.419253	-1.642063	-1.445200

C	-5.133119	-3.557048	-1.316439
O	-7.128202	-1.777799	-0.240971
O	-4.206315	-3.522154	-0.496580
O	-8.648400	-1.474780	-1.888479
C	-9.738873	-1.393533	-0.879469
H	-9.471383	-2.040095	-0.032037
H	-9.775831	-0.350097	-0.534726
C	-11.038576	-1.794956	-1.558900
H	-10.865149	-2.770305	-2.047284
O	-5.939769	-4.608257	-1.262035
C	-7.006745	-4.871532	-2.237779
H	-7.002326	-4.102262	-3.022787
H	-6.742593	-5.842100	-2.685897
C	-8.353379	-4.952869	-1.522548
H	-8.533905	-3.978514	-1.040502
C	6.450416	-0.692577	3.564084
C	6.472921	-1.560692	2.465298
C	5.355270	-0.694302	4.432336
H	5.328565	0.004075	5.270217
C	5.398722	-2.451486	2.251528
C	4.280023	-1.558063	4.204585
H	3.408221	-1.535531	4.860383
C	4.300577	-2.435829	3.116334
H	3.449776	-3.087494	2.913974
H	7.266645	0.018856	3.693202
C	7.499554	-1.396212	1.410558
C	5.340617	-3.390429	1.089401
O	7.215213	-1.514084	0.201520
O	4.410510	-3.350976	0.278648
O	8.710396	-1.116262	1.852899
C	9.755497	-0.905131	0.817603

H	9.293433	-0.372052	-0.019070
H	10.492691	-0.254578	1.301186
O	6.235834	-4.360277	0.939722
C	7.296107	-4.597510	1.931049
H	8.052119	-3.802548	1.836863
H	6.857657	-4.559830	2.940138
C	7.917791	-5.953253	1.619782
H	7.102840	-6.698194	1.639317
C	10.339684	-2.234008	0.351366
H	9.498077	-2.847414	-0.017546
C	-1.433617	-5.063593	-1.618457
H	-2.398774	-4.641468	-1.328210
H	-1.031335	-4.746090	-2.585575
C	-1.090312	-6.336467	-1.102911
C	11.281430	-1.959705	-0.832056
H	12.089223	-1.270681	-0.543416
H	11.730893	-2.900272	-1.184192
H	10.737738	-1.498166	-1.669111
C	11.043528	-2.972802	1.497123
H	10.372721	-3.128515	2.356749
H	11.404197	-3.957469	1.163004
H	11.914376	-2.398093	1.850613
C	-11.451100	-0.762221	-2.617465
H	-10.669252	-0.634089	-3.381159
H	-12.375342	-1.074592	-3.126066
H	-11.633274	0.212878	-2.138737
C	-12.121957	-1.970187	-0.480767
H	-13.073721	-2.263852	-0.947563
H	-11.845912	-2.747574	0.249099
H	-12.282260	-1.027033	0.062364
C	8.932698	-6.298649	2.721543

H	8.466916	-6.325672	3.719056
H	9.379846	-7.284983	2.531655
H	9.752347	-5.562702	2.745744
C	8.568350	-5.956762	0.229411
H	9.406949	-5.242227	0.196223
H	8.968107	-6.953113	-0.009442
H	7.849971	-5.677175	-0.553560
C	-8.352404	-6.033866	-0.433959
H	-9.320593	-6.054775	0.087357
H	-8.185048	-7.030701	-0.874608
H	-7.566957	-5.849899	0.311997
C	-9.448372	-5.190547	-2.573256
H	-9.477011	-4.384579	-3.322802
H	-9.290670	-6.146156	-3.099469
H	-10.435015	-5.237643	-2.090437
C	-2.090695	-7.053756	-0.225148
H	-1.654149	-7.902647	0.318611
H	-2.545163	-6.365686	0.499960
H	-2.895943	-7.442917	-0.870483
H	-0.488262	-6.975252	-1.753656
C	2.841191	-5.885329	1.324664
H	3.269258	-5.393734	2.213352
H	3.118896	-6.949668	1.358748
H	3.283912	-5.432570	0.428705
C	0.668961	-6.321543	2.594306
H	-0.426626	-6.275049	2.551409
H	0.972813	-7.372988	2.716878
H	0.996541	-5.761944	3.482652

148

TS_SI_DIBP SCF Done: -20628.1699334 ?.U.

Mg	-3.205115	-2.218778	0.789230

Cl	-4.995971	-1.981537	2.430755
Mg	-6.331387	-0.359172	1.235391
Cl	-8.303913	-0.018063	2.643755
Mg	-9.275169	1.889851	1.513668
Cl	-11.371149	1.691048	0.684869
Ti	-0.165687	-4.235093	-0.049818
Cl	1.672010	-4.143345	-1.679415
C	0.571360	-6.393266	0.359945
H	1.340856	-6.612919	-0.386609
C	1.137147	-5.596738	1.527772
H	0.133976	-7.316701	0.746431
H	0.608912	-5.785419	2.469464
H	2.211929	-5.817206	1.625291
H	1.185585	-4.479898	1.436979
Cl	-1.780273	-4.036370	1.754286
Mg	3.107893	-2.363694	-0.735326
Cl	4.930751	-2.292388	-2.352792
Mg	6.305856	-0.667756	-1.213004
Cl	8.357279	-0.462400	-2.533627
Mg	9.308502	1.460467	-1.435152
Cl	8.403130	3.487467	-2.381408
Mg	6.273102	3.266216	-1.289547
Cl	5.268132	5.398972	-2.042069
Mg	3.208537	5.202890	-0.855403
Cl	2.052424	7.343604	-1.449537
Mg	0.110112	6.859896	-0.186686
Cl	1.381700	5.175694	1.074222
Cl	1.237872	-2.155032	1.094857
Cl	-1.388945	-2.155829	-1.117058
Cl	11.355792	1.365978	-0.468081
Cl	-1.232702	5.173164	-1.366911

Cl	-1.805128	7.475447	1.060234
Mg	-3.043159	5.352630	0.578296
Cl	-5.064505	5.683699	1.805214
Mg	-6.170893	3.566209	1.183904
Cl	-8.256777	3.914324	2.342670
Cl	-1.751411	3.526689	1.689649
Cl	1.748772	-0.366701	-1.860113
Mg	-0.036157	-0.359214	-0.065069
Cl	-1.780150	-0.224803	1.773774
Cl	1.856395	3.388000	-1.913936
Mg	0.042858	3.306677	-0.113699
Cl	1.322777	1.531971	1.039671
Cl	-1.320405	1.547358	-1.205699
Mg	3.145519	1.435682	-0.783576
Cl	4.300096	-0.326715	0.447130
Cl	5.041496	1.457539	-2.433655
Cl	4.385387	3.363017	0.480759
Mg	-3.126467	1.587734	0.636871
Cl	-4.320155	3.515482	-0.655474
Cl	-4.963053	1.749081	2.342724
Cl	-4.363926	-0.189864	-0.493821
Cl	-7.383343	1.740910	-0.203634
Cl	7.373539	1.420275	0.210774
C	6.374361	-0.732430	3.474759
C	6.336965	-1.741432	2.504230
C	5.279697	-0.535269	4.320347
H	5.300119	0.274019	5.051760
C	5.200845	-2.573220	2.395727
C	4.149022	-1.348154	4.203567
H	3.280956	-1.178734	4.842465
C	4.109907	-2.366766	3.246776

H	3.217737	-2.984138	3.134849
H	7.239256	-0.069387	3.513428
C	7.387342	-1.772258	1.457990
C	5.057587	-3.645286	1.359663
O	7.105235	-1.908536	0.252164
O	4.138073	-3.617110	0.533407
O	8.616862	-1.639032	1.912131
C	9.716149	-1.589502	0.910489
H	9.452336	-2.257357	0.078486
H	9.761861	-0.556145	0.537170
C	11.008073	-1.980089	1.609804
H	10.833516	-2.953104	2.102405
O	5.850295	-4.710512	1.338853
C	6.907831	-4.964413	2.327040
H	6.920318	-4.168612	3.084996
H	6.621581	-5.912849	2.808054
C	8.254151	-5.101366	1.619619
H	8.456507	-4.149989	1.101388
C	-6.459148	-0.820643	-3.574046
C	-6.473591	-1.678255	-2.466847
C	-5.378727	-0.841285	-4.460382
H	-5.359089	-0.150514	-5.304757
C	-5.404103	-2.577887	-2.261770
C	-4.310590	-1.716505	-4.244661
H	-3.451406	-1.712418	-4.917243
C	-4.322265	-2.583918	-3.148181
H	-3.480797	-3.252394	-2.962330
H	-7.270550	-0.102725	-3.697359
C	-7.487967	-1.491778	-1.403376
C	-5.329021	-3.515247	-1.099382
O	-7.194215	-1.596668	-0.195915

O	-4.359619	-3.502342	-0.329771
O	-8.700589	-1.206481	-1.838549
C	-9.736620	-0.977551	-0.797633
H	-9.264255	-0.442592	0.031908
H	-10.471189	-0.324118	-1.281290
O	-6.246423	-4.454241	-0.906238
C	-7.341807	-4.682339	-1.862121
H	-8.087924	-3.882184	-1.740095
H	-6.937194	-4.645790	-2.885189
C	-7.963013	-6.033953	-1.531992
H	-7.153578	-6.783998	-1.572721
C	-10.329297	-2.297938	-0.317934
H	-9.490187	-2.915723	0.049602
C	-1.572457	-5.158619	-1.417963
H	-2.534730	-4.784933	-1.059059
H	-1.271225	-4.840200	-2.421719
C	-1.118876	-6.415002	-0.938568
C	-11.260485	-2.009593	0.870457
H	-12.065371	-1.316564	0.583597
H	-11.713689	-2.944963	1.231523
H	-10.707517	-1.546905	1.700699
C	-11.047509	-3.037888	-1.453882
H	-10.386041	-3.199837	-2.319480
H	-11.409790	-4.019348	-1.112130
H	-11.918954	-2.460207	-1.800962
C	11.403838	-0.938786	2.666535
H	10.614412	-0.812134	3.422576
H	12.325455	-1.242817	3.184887
H	11.584142	0.035049	2.184693
C	12.105120	-2.156497	0.545579
H	13.052006	-2.445194	1.025166

H	11.840942	-2.937691	-0.184603
H	12.269245	-1.214688	0.001151
C	-9.009673	-6.374498	-2.605391
H	-8.570816	-6.407841	-3.614847
H	-9.458998	-7.356862	-2.400632
H	-9.823708	-5.632310	-2.609733
C	-8.575654	-6.031063	-0.124453
H	-9.411428	-5.314333	-0.071144
H	-8.971297	-7.025520	0.128661
H	-7.835761	-5.750552	0.637959
C	8.232831	-6.223346	0.573486
H	9.202950	-6.287982	0.059327
H	8.038898	-7.197913	1.051559
H	7.455856	-6.050136	-0.183847
C	9.341422	-5.321333	2.682159
H	9.384067	-4.488278	3.400757
H	9.163178	-6.253117	3.243369
H	10.328119	-5.406169	2.204639
C	-0.475461	-7.376067	-1.913463
H	0.001298	-8.238047	-1.426745
H	-1.263796	-7.759374	-2.581877
H	0.270648	-6.857834	-2.533983
H	-1.749538	-6.869006	-0.169617

124

III_DEP SCF Done: -20313.5598110 □.U.

Mg	-3.239405	-2.469453	0.921676
Cl	-5.103077	-2.180131	2.443437
Mg	-6.400983	-0.670083	1.069420
Cl	-8.448176	-0.327110	2.345671
Mg	-9.385951	1.522842	1.106327
Cl	-11.409644	1.170307	0.153170

Ti	-0.018394	-4.193377	0.157839
Cl	1.624108	-4.074751	-1.669582
C	-0.099212	-6.268796	0.182924
H	-0.904329	-6.427873	0.917559
C	1.251761	-6.215724	0.853836
H	1.528565	-5.151703	1.166842
H	1.304735	-6.759541	1.808408
H	2.079950	-6.501525	0.193550
Cl	-1.651646	-3.999270	1.998624
Mg	3.221947	-2.503582	-0.646430
Cl	5.046713	-2.336178	-2.228515
Mg	6.375504	-0.760764	-0.966124
Cl	8.403348	-0.494336	-2.291259
Mg	9.365347	1.421311	-1.173598
Cl	8.438803	3.453152	-2.081622
Mg	6.278999	3.173456	-1.043250
Cl	5.247981	5.282695	-1.804131
Mg	3.156810	5.042376	-0.679312
Cl	1.986072	7.151751	-1.341972
Mg	0.000853	6.634467	-0.164641
Cl	1.251663	4.994193	1.175988
Cl	1.362792	-2.389741	1.296903
Cl	-1.366514	-2.457992	-1.015869
Cl	11.406128	1.124452	-0.238123
Cl	-1.248601	4.904101	-1.388132
Cl	-1.985679	7.231607	0.971664
Mg	-3.157903	5.082499	0.447805
Cl	-5.256268	5.404334	1.539327
Mg	-6.289281	3.253640	0.910619
Cl	-8.459509	3.600749	1.908777
Cl	-1.886755	3.304848	1.659915

Cl	1.806634	-0.520787	-1.660498
Mg	-0.001074	-0.581845	0.093482
Cl	-1.824814	-0.413793	1.819244
Cl	1.877935	3.186961	-1.756616
Mg	-0.000872	3.080832	-0.038775
Cl	1.280250	1.349576	1.206802
Cl	-1.277658	1.265017	-1.159117
Mg	3.176697	1.287862	-0.544919
Cl	4.332515	-0.501132	0.671335
Cl	5.073871	1.350954	-2.187675
Cl	4.338239	3.239895	0.708216
Mg	-3.183768	1.323920	0.574033
Cl	-4.330510	3.190841	-0.819181
Cl	-5.102108	1.502514	2.181981
Cl	-4.329955	-0.538906	-0.537106
Cl	-7.376043	1.361438	-0.489052
Cl	7.376880	1.359227	0.457125
C	6.245865	-1.199518	3.841565
C	6.269577	-2.090109	2.757917
C	5.136743	-1.148141	4.688804
H	5.116616	-0.430612	5.510463
C	5.171094	-2.952317	2.543698
C	4.044363	-1.989308	4.462960
H	3.163726	-1.935292	5.104931
C	4.064364	-2.893324	3.396719
H	3.208551	-3.543393	3.212067
H	7.080673	-0.511151	3.974911
C	7.338126	-1.941794	1.737674
C	5.096037	-3.936467	1.416488
O	7.103168	-2.039538	0.517452
O	4.247097	-3.834450	0.524655

O	8.524898	-1.656707	2.240477
C	9.607178	-1.270368	1.291306
H	9.132871	-0.814764	0.417986
H	10.173972	-0.508827	1.835565
C	10.442914	-2.461544	0.882702
H	9.857972	-3.166788	0.275995
O	5.885570	-5.003781	1.387280
C	6.739767	-5.390305	2.524609
H	6.523220	-4.745375	3.388015
H	6.420323	-6.415888	2.756976
C	8.196028	-5.335196	2.112041
H	8.526139	-4.304545	1.935226
C	-6.161945	-1.454289	-3.724622
C	-6.241652	-2.232290	-2.561842
C	-5.033771	-1.531568	-4.545133
H	-4.966605	-0.900956	-5.433008
C	-5.184959	-3.110259	-2.234673
C	-3.982359	-2.390023	-4.212220
H	-3.088981	-2.435727	-4.837051
C	-4.057770	-3.179700	-3.061088
H	-3.233131	-3.839274	-2.787836
H	-6.966087	-0.752262	-3.946434
C	-7.333713	-1.977859	-1.591219
C	-5.170341	-3.963713	-1.006436
O	-7.139678	-1.995850	-0.359331
O	-4.255342	-3.871550	-0.179391
O	-8.501039	-1.718205	-2.148199
C	-9.617934	-1.325571	-1.245224
H	-9.186666	-0.790858	-0.394587
H	-10.212716	-0.630057	-1.844809
O	-6.079522	-4.910903	-0.808548

C	-7.137300	-5.197146	-1.792130
H	-7.964480	-4.497602	-1.599913
H	-6.748667	-5.022082	-2.806300
C	-7.563667	-6.634238	-1.576811
H	-7.922149	-6.784827	-0.549489
C	-10.391046	-2.540152	-0.782735
H	-10.786903	-3.115487	-1.631883
C	-0.406991	-7.059968	-1.100943
H	-0.932195	-7.982315	-0.786780
C	-1.404486	-6.247724	-1.961004
H	-0.906053	-5.373399	-2.414843
H	-1.815265	-6.853931	-2.783911
H	-2.251006	-5.889321	-1.349153
C	0.812051	-7.496605	-1.920373
H	1.465192	-8.169685	-1.344294
H	0.483997	-8.044090	-2.817469
H	1.410508	-6.637144	-2.252593
H	-6.728613	-7.323300	-1.764205
H	-8.380915	-6.879134	-2.271274
H	11.269952	-2.081512	0.264876
H	10.865738	-2.980294	1.754853
H	-11.235720	-2.185065	-0.174368
H	-9.765011	-3.187915	-0.152119
H	8.365187	-5.920058	1.197719
H	8.817122	-5.756149	2.916809

148

III__DIBP SCF Done: -20628.1981989 ⍰.U.

Mg	3.255058	-2.156539	-0.950709
Cl	5.050205	-1.851180	-2.554518
Mg	6.377305	-0.324053	-1.229797
Cl	8.429146	0.028758	-2.525808

Mg	9.365648	1.846276	-1.225450
Cl	11.405119	1.611072	-0.279595
Ti	0.057086	-3.901388	-0.089890
Cl	-1.551190	-3.775130	1.768135
C	0.149762	-5.976477	-0.096586
H	0.944813	-6.136121	-0.842143
C	-1.211407	-5.936848	-0.747838
H	-1.500668	-4.876981	-1.063343
H	-1.274934	-6.487305	-1.697942
H	-2.027596	-6.224369	-0.073550
Cl	1.652312	-3.715056	-1.966291
Mg	-3.180626	-2.218342	0.771696
Cl	-4.977674	-2.054054	2.390082
Mg	-6.340481	-0.510573	1.127603
Cl	-8.358631	-0.213624	2.484358
Mg	-9.333944	1.672103	1.310940
Cl	-8.399374	3.708976	2.206921
Mg	-6.254472	3.428095	1.156934
Cl	-5.226821	5.551043	1.893379
Mg	-3.156102	5.320135	0.734872
Cl	-1.985674	7.439911	1.366803
Mg	-0.018463	6.926030	0.159541
Cl	-1.284155	5.270771	-1.150371
Cl	-1.357928	-2.117811	-1.217579
Cl	1.421708	-2.150193	1.036576
Cl	-11.384031	1.510272	0.370497
Cl	1.261512	5.208328	1.372031
Cl	1.945253	7.526838	-1.012314
Mg	3.137685	5.387287	-0.490055
Cl	5.219059	5.717156	-1.606533
Mg	6.270768	3.570409	-0.983866

Cl	8.417807	3.926844	-2.010489
Cl	1.855491	3.600471	-1.678365
Cl	-1.755025	-0.225344	1.735459
Mg	0.016780	-0.294022	-0.057659
Cl	1.795466	-0.118960	-1.827615
Cl	-1.850782	3.479075	1.803105
Mg	-0.002915	3.370426	0.053300
Cl	-1.296772	1.625073	-1.157721
Cl	1.304445	1.570101	1.159214
Mg	-3.158902	1.562337	0.628696
Cl	-4.322299	-0.245751	-0.548754
Cl	-5.026754	1.625856	2.304659
Cl	-4.352880	3.496877	-0.622456
Mg	3.171242	1.628049	-0.613612
Cl	4.343684	3.496643	0.761988
Cl	5.067309	1.827162	-2.261097
Cl	4.349560	-0.223601	0.465061
Cl	7.389438	1.638397	0.370996
Cl	-7.361415	1.564045	-0.311283
C	-6.318267	-0.995996	-3.685256
C	-6.293013	-1.871052	-2.588659
C	-5.239914	-0.941647	-4.570699
H	-5.258409	-0.236526	-5.403062
C	-5.175903	-2.716213	-2.400583
C	-4.127115	-1.761925	-4.368340
H	-3.268572	-1.703435	-5.039248
C	-4.098029	-2.650928	-3.289678
H	-3.226217	-3.285184	-3.125052
H	-7.166521	-0.320674	-3.798662
C	-7.323877	-1.723493	-1.531610
C	-5.050805	-3.694984	-1.271367

O	-7.046641	-1.850247	-0.321697
O	-4.212751	-3.553621	-0.375578
O	-8.524328	-1.407342	-1.983086
C	-9.581412	-1.143604	-0.972489
H	-9.099233	-0.715110	-0.088470
H	-10.226023	-0.391108	-1.440762
C	-10.319577	-2.425701	-0.606449
H	-9.556311	-3.151578	-0.274985
O	-5.774442	-4.807991	-1.254754
C	-6.595902	-5.229610	-2.400823
H	-6.290834	-4.672706	-3.298987
H	-6.333138	-6.290646	-2.527404
C	-8.085293	-5.071396	-2.118060
H	-8.301721	-3.992702	-2.055202
C	6.421521	-1.098783	3.541144
C	6.405608	-1.907343	2.398119
C	5.338123	-1.116302	4.424632
H	5.343634	-0.462909	5.298498
C	5.300975	-2.752567	2.154776
C	4.238880	-1.942006	4.173298
H	3.382057	-1.938390	4.848912
C	4.220211	-2.763261	3.041495
H	3.359655	-3.400349	2.832115
H	7.260308	-0.419950	3.698462
C	7.436056	-1.711117	1.347071
C	5.223000	-3.643924	0.958839
O	7.131414	-1.655287	0.142121
O	4.313711	-3.543756	0.127958
O	8.667871	-1.591045	1.802117
C	9.741259	-1.233861	0.832449
H	9.278163	-0.711039	-0.013068

H	10.375233	-0.535761	1.389748
O	6.106431	-4.621961	0.790923
C	7.066258	-4.977614	1.852941
H	7.830742	-4.190367	1.921275
H	6.528628	-5.036937	2.813403
C	7.690694	-6.313056	1.472204
H	8.130157	-6.188440	0.467158
C	10.499444	-2.466608	0.351282
H	10.828676	-3.027218	1.245408
C	6.638729	-7.428908	1.422237
H	5.835040	-7.189012	0.711750
H	7.096218	-8.379678	1.111869
H	6.187195	-7.582135	2.416852
C	8.819590	-6.628122	2.466080
H	8.429344	-6.726111	3.492402
H	9.304766	-7.579173	2.204006
H	9.592530	-5.843965	2.467075
C	-8.866495	-5.643092	-3.311604
H	-8.605849	-5.136767	-4.254163
H	-8.667232	-6.720248	-3.433560
H	-9.946424	-5.519579	-3.151606
C	-8.490837	-5.729822	-0.794665
H	-9.568992	-5.601555	-0.619694
H	-8.278552	-6.811234	-0.814385
H	-7.949773	-5.291619	0.055744
C	-11.248567	-2.136258	0.583835
H	-11.797375	-3.047925	0.864021
H	-10.675977	-1.793317	1.458096
H	-11.975354	-1.349165	0.334676
C	-11.083043	-2.996262	-1.808136
H	-10.429344	-3.132749	-2.682648

H	-11.529072	-3.971184	-1.559561
H	-11.900654	-2.318329	-2.100801
C	9.631687	-3.375335	-0.532863
H	10.210669	-4.249979	-0.864924
H	8.736148	-3.744284	-0.014078
H	9.288812	-2.827190	-1.423554
C	11.742362	-1.986170	-0.420055
H	12.389641	-1.346757	0.196148
H	12.326467	-2.851250	-0.767628
H	11.446529	-1.393968	-1.299190
C	0.480681	-6.757983	1.187586
H	1.003591	-7.680997	0.871334
C	1.488245	-5.937408	2.027564
H	0.994072	-5.061470	2.482609
H	1.912552	-6.537350	2.848132
H	2.324969	-5.580018	1.402053
C	-0.724252	-7.192787	2.028657
H	-1.384316	-7.871302	1.466993
H	-0.380632	-7.733615	2.923952
H	-1.319877	-6.332780	2.364590

i. Patthamasang, S.; Jongsomjit, B.; Praserthdam, P. Efect of EtOH/$MgCl_2$ molar ratios on the catalytic properties of $MgCl_2$-SiO_2/$TiCl_4$ Ziegler–Natta catalyst for ethylene polymerization. *Molecules* **2011**, *16*, 8332–8342.

ii. Dechant, J. *Polymer Handbook*, 3rd ed.; Brandrup, J., Immergut, E.H., Eds.; John Wiley & Sons: Hoboken, NJ, USA, 1989. ISBN 0-471-81244-7.

iii. Müller, A.J.; Arnal, M.L. Thermal fractionation of polymers. *Prog. Polym. Sci.* **2005**, *30*, 559–603.

iv. Müller, A.J.; Lorenzo, A.T.; Arnal, M.L. Recent Advances and Applications of "Successive Self-Nucleation and Annealing"(SSA) High Speed Thermal Fractionation. *Macromol. Symp.* **2009**, *277*, 207–214.

v. Wunderlich, B.; Czornyj, G. GA study of equilibrium melting of polyethylene. *Macromolecules* **1977**, *10*, 906–913.

vi. Bond, E.B.; Spruiell, J.E.; Lin, J.S. A WAXD/SAXS/DSC study on the melting behavior of Ziegler–Natta and metallocene catalyzed isotactic polypropylene. *J. Polym. Sci. Part B Polym. Phys.* **1999**, *37*, 3050–3064.

vii. Iijima, M.; Strobl, G. Isothermal crystallization and melting of isotactic polypropylene analyzed by time-and temperature-dependent small-angle X-ray scattering experiments. *Macromolecules* **2000**, *33*, 5204–5214.

[viii]. A Kebritchi, A.; Nekoomanesh, M.; Mohammadi, F.; Khonakdar, H.; Wagenknecht, U. Thermal behavior of ethylene/1-octene copolymer fractions at high temperatures: Effect of hexyl branch content. *Polyolefins J.* **2019**, *6*, 127–138.

[ix]. Torabi, S.R.; Fazeli, N. A rapid quantitative method for determination of short chain branching content and branching distribution index in LLDPEs by DSC. *Polym. Test.* **2009**, *28*, 866–870.
